# DOES THE ENERGETIC COST OF STRESS ACCELERATE BIOLOGICAL AGING AND SHORTEN LIFESPAN?

**DOI:** 10.1093/geroni/igac059.1145

**Published:** 2022-12-20

**Authors:** Martin Picard

**Affiliations:** Columbia University, New York, New York, United States

## Abstract

In humans, chronic activation of cellular stress responses predict functional decline, accelerate aging, and increase mortality, but the cellular basis for the stress-aging cascade remains unclear. Here we induced chronic stress in primary human fibroblasts from multiple donors with constant i) ATP-synthase inhibition (Oligomycin-1nM) or i) glucocorticoid stimulation (Dexamethasone-100nM) cultured for up to 10 months. Stressors triggered mtDNA instability and activated integrated stress responses resulting in both transcriptional activation and secretion of cytokines and metabokines. In parallel, chronic stress increased cellular energy expenditure or the “cost of living” by 62–108% (Ps<0.001). Thus, chronically stressed cells considerably expend more energy to undergo each cell division. This severe state of hypermetabolism led to faster rates of telomere shortening and of genome-wide DNA methylation-based epigenetic aging monitored across the cellular lifespan, reflecting mito-nuclear signaling. This accelerated aging phenotype culminated in 20–40% fewer maximal cell divisions (i.e., Hayflick limit). Based on findings that hypermetabolism and increased energy flux through mitochondria may shorten lifespan, we pharmacologically inhibited carbon entry (glutamine, pyruvate, long-chain fatty acids) into the Krebs cycle across the entire cellular lifespan. While this manipulation successfully decreased OxPhos activity, it increased glycolysis-derived ATP synthesis and total energy expenditure, exacerbating the accelerated aging phenotype. Combined, our longitudinal bioenergetic and multi-omic profiling of primary human cells show that chronic heterotypic stressors converge on an acceleration of metabolism (i.e., hypermetabolism), and commensurately accelerate the progression of multiple aging hallmarks. These findings also implicate long-term mito-nuclear signaling in the stress-aging cascade in a human model.

